# Social and non‐social reward processing in subclinical depression

**DOI:** 10.1002/pchj.691

**Published:** 2023-10-31

**Authors:** Yu Chen, Yi‐hang Huang, Ling‐ling Wang, Hui‐xin Hu, Min‐yi Chu, Simon S. Y. Lui, Raymond C. K. Chan

**Affiliations:** ^1^ Translational Neuropsychology and Applied Cognitive Neuroscience Laboratory, Shanghai Mental Health Center Shanghai Jiao Tong University School of Medicine Shanghai China; ^2^ Neuropsychology and Applied Cognitive Neuroscience Laboratory, CAS Key Laboratory of Mental Health Institute of Psychology Beijing China; ^3^ Department of Psychology University of Chinese Academy of Sciences Beijing China; ^4^ Department of Psychiatry, School of Clinical Medicine The University of Hong Kong Hong Kong Administrative Region China

**Keywords:** depressive symptoms, reward, social reward

## Abstract

This study applied two incentive delay tasks involving social and non‐social incentive types to 76 pairs of participants with high and low depressive symptoms. The results suggest that higher levels of depressive symptoms are correlated with abnormalities in social and non‐social reward processing even in the healthy populations.

Impaired reward processing is believed to contribute to anhedonia and avolition symptoms in psychiatric disorders (Tomarken & Keener, 1998). Patients with major depressive disorder (MDD) manifest depressive symptoms, such as lack of energy, lack of pleasurable experiences, and loss of interest, which are related to the neuropsychological underpinning of impaired reward processing. Reward processing refers to an individual's motivation to seek out incentive/reward, engage in reward‐seeking, and modulate behavior in response to reward cues, and the incentive/reward involved may be of monetary and social nature. Recent empirical evidence suggested that MDD patients may show abnormalities in both social and non‐social reward processing (Klawohn et al., 2021). However, MDD is a treatable psychiatric disorder, and MDD patients often receive psychotropic medications, which might have affected the administration of the neuropsychological paradigm. Research on subclinical samples with attenuated depressive symptomatology that does not justify a diagnosis of MDD is a method to explore the association of MDD with impaired reward processing. However, few studies have examined social and non‐social reward processing in subclinical samples with depressive symptoms in the general population.

This study aimed to study social and non‐social reward processing in people with subclinical MDD symptoms. Participants who scored above or equal to 10 points on the Patient Health Questionnaire (PHQ‐9; Kroenke & Spitzer, 2002) were classified as individuals with high levels of depressive symptoms, whereas participants who scored below 10 points on the PHQ‐9 were classified as individuals with low levels of depressive symptoms. Our sample comprised 76 college students (38.2% male; mean age = 20.39 years, *SD* = 1.83 years) with high levels of depressive symptoms (PHQ‐9 score: mean = 13.30, *SD* = 3.41) and 73 college students (24.7% male, mean age = 20.37 years, *SD* = 1.71 years) with low levels of depressive symptoms (PHQ‐9 score: mean = 4.08, *SD* = 2.60). Participants completed several questionnaires online, that is, the PHQ‐9, the Temporal Experience of Pleasure Scale (TEPS; Chan et al., 2012), the Motivation and Pleasure Scale‐Self‐Report (MAP_SR; Wang et al., 2020), and two online behavioral tasks, that is, the Monetary Incentive Delay Task (MID; Knutson et al., 2000), and the Social Incentive Delay Task (SID; Zhang et al., 2022). In the MID and SID tasks, participants first viewed a hint (triangle, square, circle) indicated three conditions (reward, punishment, or neutral) and possible feedback. Then participants were asked to make a quick response to press the button. In the MID task, they received monetary reward if they won in reward conditions, and had monetary loss if they lost in the punishment condition. In the SID task, they received a ‘thumbs‐up’ emoji or ‘thumbs‐down’ emoji as social reward and punishment feedback. We measured the number of hits and the response time (RT) in both tasks. This study was approved by the Ethics Committees of the Shanghai Mental Health Centre and the Institute of Psychology, Chinese Academy of China. [Corrections added on 23 November 2023, after first online publication: The *SD* value of 76 college students and the *mean* and *SD* value of PHQ‐9 score of the above paragraph have been amended.]

The results (see Table 1) indicated that the two groups differed in the TEPS (total score: *t*(147) = −3.26, *p* < .05; Anticipatory subscale: *t*(147) = −2.952, *p* < .05; Consummatory subscale: *t*(147) = −3.01, *p* < .05) and the MAP‐SR (*t*(147) = −6.05, *p* < .001). The 2 (Task Type: MID, SID) × 2 (Group: depressed, controls) × 3 (Condition: reward, neutral, punishment) repeated‐measure analysis of variance (ANOVA) showed that the Task Type (*F*(1, 147) = 63.217, *p* < .001, *η* ^*2*^ *_p_* = .301) and Condition (*F*(2, 294) = 72.694, *p* < .001, *η* ^*2*^ *_p_* = .331) main effects were both significant for RT. Specifically, participants had shorter RTs for the MID (*M* = 0.229 s) than the SID (*M* = 0.253 s) task, and for the reward (*M* = 0.233 s) than the neutral (*M* = 0.250 s) and punishment (*M* = 0.240 s) conditions. The Group main effect for RT was significant (*F*(1, 147) = 6.641, *p* < .05, *η* ^2^ *_p_* = .043), suggesting that controls had shorter RTs (*M* = 0.234 s) than the depressed group (*M* = 0.248 s). The Task‐Type‐by‐Condition interaction was significant (*F*(2, 294) = 46.414, *p* < .001, *η* ^2^ *_p_* = .24), but not the Group‐by‐Task‐Type, Group‐by‐Condition, or three‐way interaction (*p*s > .05). Regarding the number of hits, the Condition main effect was significant (*F*(2, 294) = 5.046, *p* < .05), but not the Task Type or Group main effects (*p*s > .05). Likewise, the Task‐Type‐by‐Condition interaction was significant (*F*(2, 294) = 5.271, *p* < .05, *η* ^*2*^ *_p_* = .035), but not the Group‐by‐Task‐Type, Group‐by‐Condition, or three‐way interaction (*p*s > .05). [Corrections added on 23 November 2023, after first online publication: The “*t*” value of TEPS, Anticipatory subscale and MAP‐SR and the “*F*” value of Task Type and Condition of the ANOVA of the above paragraph have been amended.]

**TABLE 1:** MID and SID performances between participants with high and low levels of self‐reported depression

			*M* (*SD*)	SS	df	*F*	*p*	*η* ^2^ *_p_*
Measurement: RTs								
Task	MID		0.229 (0.003)	0.13	1	63.217	.000	.301
	SID		0.253 (0.003)					
Condition	Reward		0.233 (0.003)	0.048	2	72.694	.000	.331
	Punishment		0.24 (0.003)					
	Neutral		0.25 (0.003)					
Group	Depressed		0.248 (0.004)	0.045	1	6.641	.011	.043
	Controls		0.234 (0.004)					
Task × Condition	MID	Reward	0.217 (0.003)	0.027	2	46.414	.000	.24
		Punishment	0.223 (0.003)					
		Neutral	0.246 (0.003)					
	SID	Reward	0.248 (0.004)					
		Punishment	0.257 (0.004)					
		Neutral	0.255 (0.004)					
Group × Condition	Depressed	Reward	0.24 (0.004)	0.001	2	1.209	.300	.008
		Punishment	0.248 (0.004)					
		Neutral	0.256 (0.004)					
	Controls	Reward	0.225 (0.004)					
		Punishment	0.232 (0.004)					
		Neutral	0.245 (0.004)					
Group × Task	Depressed	MID	0.235 (0.004)	0.001	1	0.499	0.481	0.003
		SID	0.261 (0.005)					
	Controls	MID	0.223 (0.004)					
		SID	0.245 (0.005)					
Measurement: Number of hits								
Task	MID		11.447 (0.197)	0.038	1	0.004	.950	0
	SID		11.46 (0.128)					
Condition	Reward		11.632 (0.162)	36.821	2	5.046	.007	.033
	Punishment		11.56 (0.151)					
	Neutral		11.17 (0.161)					
Group	Depressed		11.632 (0.181)	28.206	1	1.879	.172	.013
	Controls		11.276 (0.185)					
Task × Condition	MID	Reward	11.763 (0.25)	36.139	2	5.271	.006	.035
		Punishment	11.7 (0.238)					
		Neutral	10.879 (0.246)					
	SID	Reward	11.5 (0.166)					
		Punishment	11.42 (0.168)					
		Neutral	11.461 (0.157)					
Group × Condition	Depressed	Reward	11.757 (0.226)	1.693	2	0.232	.793	.002
		Punishment	11.737 (0.211)					
		Neutral	11.401 (0.226)					
	Controls	Reward	11.507 (0.231)					
		Punishment	11.384 (0.215)					
		Neutral	10.938 (0.23)					
Group × Task	Depressed	MID	11.671 (0.276)	1.89	1	0.197	.658	.001
		SID	11.592 (0.179)					
	Controls	MID	11.224 (0.281)					
		SID	11.329 (0.182)					

Abbreviations: MID, monetary incentive delay task; RTs, response times; SID, social incentive delay task; SS, sum of squares. [Correction added on 23 November 2023, after first online publication: The values of the Group × Task under Measurement: RTs in columns *F*, *p* and *η* ^2^ *_p_* have been amended.]

Together, our findings suggested that college students with depressive symptoms exhibited a mild degree of impaired reward processing, resulting in the longer RTs than controls when responding to stimuli of social and non‐social (monetary) nature. Therefore, participants with high levels of depressive symptoms exhibited reduced pleasure on TEPS scores and MAP‐SR scores compared to participants with low levels of depressive symptoms. Although a previous study (Xie et al., 2014) reported that college students with elevated social anhedonia exhibited altered reward processing for social affective incentives but not monetary incentives, we did not find any significant Task‐Type‐by‐Group interaction. As such, it is plausible that unlike people with social anhedonia, our sample with subclinical depressive symptoms did not show any “domain‐specific deficit” affecting mainly social incentives. In fact, depressive symptoms appeared to result in generalized reduction of anticipatory pleasure, not specific to the social domain. One limitation of the study is that our participants were college students with a narrow age range (mean age = 20.38 years). Future studies should be conducted across the lifespan. To conclude, our preliminary study provided further evidence to support subtle impairments in reward processing on both social and non‐social stimuli in subclinical samples with depressive symptoms, and further implicate the role of impaired reward processing as a putative neuropsychological underpinning for MDD.

## CONFLICT OF INTEREST STATEMENT

The authors declare there are no conflicts of interest.

## ETHICS STATEMENT

The study was approved by the Ethics Committee of the Institute of Psychology, Chinese Academy of Sciences. All participants gave written informed consent.
